# Evaluation of coronary blood flow velocity during cardiac arrest with circulation maintained through mechanical chest compressions in a porcine model

**DOI:** 10.1186/1471-2261-11-73

**Published:** 2011-12-19

**Authors:** Henrik Wagner, Bjarne Madsen Hardig, Stig Steen, Trygve Sjoberg, Jan Harnek, Goran K Olivecrona

**Affiliations:** 1 Department of Cardiology, Skane University Hospital, Lund, Lund University, SE-221 85 Lund, Sweden; 2 Jolife AB, Ideon Science Park, SE-223 70, Lund, Sweden; 3 Department of Thoracic Surgery, Skane University Hospital, Lund, Lund University, SE-221 85 Lund, Sweden

## Abstract

**Background:**

Mechanical chest compressions (CCs) have been shown capable of maintaining circulation in humans suffering cardiac arrest for extensive periods of time. Reports have documented a visually normalized coronary blood flow during angiography in such cases (TIMI III flow), but it has never been actually measured. Only indirect measurements of the coronary circulation during cardiac arrest with on-going mechanical CCs have been performed previously through measurement of the coronary perfusion pressure (CPP). In this study our aim was to correlate average peak coronary flow velocity (APV) to CPP during mechanical CCs.

**Methods:**

In a closed chest porcine model, cardiac arrest was established through electrically induced ventricular fibrillation (VF) in eleven pigs. After one minute, mechanical chest compressions were initiated and then maintained for 10 minutes upon which the pigs were defibrillated. Measurements of coronary blood flow in the left anterior descending artery were made at baseline and during VF with a catheter based Doppler flow fire measuring APV. Furthermore measurements of central (thoracic) venous and arterial pressures were also made in order to calculate the theoretical CPP.

**Results:**

Average peak coronary flow velocity was significantly higher compared to baseline during mechanical chests compressions and this was observed during the entire period of mechanical chest compressions (12 - 39% above baseline). The APV slowly declined during the 10 min period of mechanical chest compressions, but was still higher than baseline at the end of mechanical chest compressions. CPP was simultaneously maintained at > 20 mmHg during the 10 minute episode of cardiac arrest.

**Conclusion:**

Our study showed good correlation between CPP and APV which was highly significant, during cardiac arrest with on-going mechanical CCs in a closed chest porcine model. In addition APV was even higher during mechanical CCs compared to baseline. Mechanical CCs can, at minimum, re-establish coronary blood flow in non-diseased coronary arteries during cardiac arrest.

## Background

Mechanical chest compression (CC) devices used during cardiac arrest in the cardiac catheterization laboratory (cath-lab) have been shown capable of producing adequate coronary perfusion pressures (CPP) which is well correlated to normal coronary blood flow, Thrombolysis In Myocardial Infarction-flow (TIMI-flow) III [1]. TIMI III flow in conjunction with mechanical CCs during cardiac arrest in patients has also been reported by Bonnemeier et al. [2,3]. TIMI-flow is commonly used in the cardiac cath-lab to evaluate coronary blood flow, however intracoronary Doppler blood flow measurements were not performed in these studies to correlate with the visual TIMI-flow [1-3]. Cardiac arrest (CA) due to ventricular fibrillation (VF) has frequently been used in animal cardiopulmonary resuscitation (CPR) models for measuring CPP, which is done by calculating the pressure gradient between the aorta and the right atrium during the end of the decompression phase [4-6]. Studies in animals and in humans have demonstrated a positive correlation between CPP and return of spontaneous circulation (ROSC) [5-9], however, little is known about the relationship between CPP and directly measured intracoronary blood flow velocity during mechanical CCs or manual CCs. Measurements of intracoronary blood flow is performed with a Doppler flow wire and the method has previously been used to measure coronary blood flow before and after percutaneous coronary intervention (PCI) in patients with stable circulation [10-12] or when correlating other modalities for measuring coronary blood flow [13,14]. One study used a cardio pulmonary bypass system to mimic a low flow state and an ultrasonic flow probe inserted in the left anterior descending artery (LAD) in a porcine open chest model [15], thus this method is well documented.

LUCAS™ (Jolife AB, Lund, Sweden) is a mechanical CCs device that delivers compressions according to international guidelines for resuscitation [16,17] and it has been shown to produce significantly higher mean arterial blood pressure, coronary perfusion pressure [9] and increases cerebral blood flow compared to manual CCs over extended periods of CA in pig models [18]. During cardiac/circulatory arrest in human as well as pig studies, using manual CCs over extended periods of time, blood pressure has been shown to successively decline and finally result in a low flow or no flow situation most often due to impaired manual CCs or development of stone heart [3,6]. This negative downward spiral will further reduce the possibility to obtain return of ROSC [8].

The aim of this study was to explore the correlation between CPP and Doppler blood flow in the LAD during mechanical CCs in an experimental animal model. To our knowledge this has not been done before.

## Methods

### Animal care

Eleven Swedish-bred specific (Swedish Landrace) pathogen free pigs with a mean weight of 31 kilo (range 28 - 31 kg) were used. Temperature of the pigs at VF was 37.9°C ± 0.7°C. The animals received humane care in compliance with *The Guide for the Care and Use of Laboratory Animals*, published by the national institute of health (NIH publication 85 - 23, revised 1985) and the European convention for the Protection of Vertebrate Animals used for experimental and Other Scientific Purposes (1986). The institutional Review Board for animal experimentation at Lund University, Sweden, approved the experimental protocols.

### Anesthesia and preparation

The pigs had free access to water but were not allowed to eat on the day of experiment. They were anaesthetized with an induction dose of intramuscular ketamine (30 mg/kg). Sodium thiopental (5-8 mg/kg) and atropine (0.015 mg/kg) were given intravenously before tracheotomy. Anesthesia and muscular paralysis were maintained with continuous infusion of 10 ml/h of a 0.9% saline solution containing ketamine (16 mg/ml) and pancuronium (0.6 mg/ml).

### Ventilator settings and instrumentation

A Boussignac ET tube, 7 mm internal diameter (Laboratories Pharamceutiques VYGON, Ecouen, France) was used as an ordinary endotracheal tube for ventilation. After tracheothomy it was connected to a Servo Ventilator 300 (Servo Ventilator 300, Siemens, Solna, Sweden) using pressure-regulated (max 30 cmH_2_O = 23 mmHg) and volume controlled intermittent positive pressure ventilation (IPPV). Normo-ventilation (end-tidal CO_2 _around 5.3 kPa = 40 mmHg) was obtained by using a tidal volume of 8 ml/kg body weight, 20 breaths/min, a PEEP of 5 cmH_2_O (6 mmHg) and a FiO_2 _of 0.21. End tidal CO_2 _was measured by CO_2_SMO Plus respiratory Profile Monitor (Model 8100; Novametrix Medical Systems Inc., Wallingsford, CT, USA) with a CO_2 _sensor (REF 6719) connected to the proximal end of the Boussignac tube.

For monitoring of aortic pressure and central venous pressure, two catheters (Secalon-T-over-needle catheter, 16G/1.70/130 mm) were introduced via direct puncture of the right carotid artery and the right jugular vein respectively, in order to avoid ligation of the artery. The tip of the arterial catheter was inserted into the thoracic aorta and the central venous catheter was placed with the tip in the right atrium. The fluid filled catheters were connected via short tubes to pressure transducers.

A temperature probe was placed in the esophagus and electrocardiogram (ECG) was obtained by three electrodes glued to the chest. The following variables were continuously sampled (100 - 500 Hz) to a computer supplied with a data acquisition system (Testpoint, Capital Equipment Corporation, Billerica, Massachusetts): body temperature, ECG, intra thoracic arterial pressure (AP), right atrial pressure (CVP), CPP, End Tidal CO_2 _(ETCO_2_).

A 6 F introducer sheath (Boston Scientific Scimed, Maple Grove, MN, USA) was inserted into the surgically exposed left carotid artery and a 6F JL 3.5 Wiseguide™ (Boston Scientific Scimed, Maple Grove, MN, USA) catheter was then inserted through the introducer for placement of the tip in the left main coronary artery and 10,000 IU of Heparin was administered. The catheter was used to place a 0.014-inch, 12 MHz pulsed Doppler flow velocity transducer (FloWire^® ^Volcano Inc., San Diego CA) into the mid-portion of the LAD. Continuous coronary velocity flow profiles were displayed and recorded using the Doppler flow wire connected to a FloMap monitor (Cardio metrics, Mountain View, CA). Coronary flow is in this system measured as the average peak velocity (APV), in which APV is the time-averaged value of the instantaneous peak velocity samples over the last 2 cardiac cycles in centimeters per second. All radiological procedures were performed in an experimental catheterization laboratory, (GE Healthcare, Chalfont St Giles, UK).

### Experimental protocol

**Figure 1:**
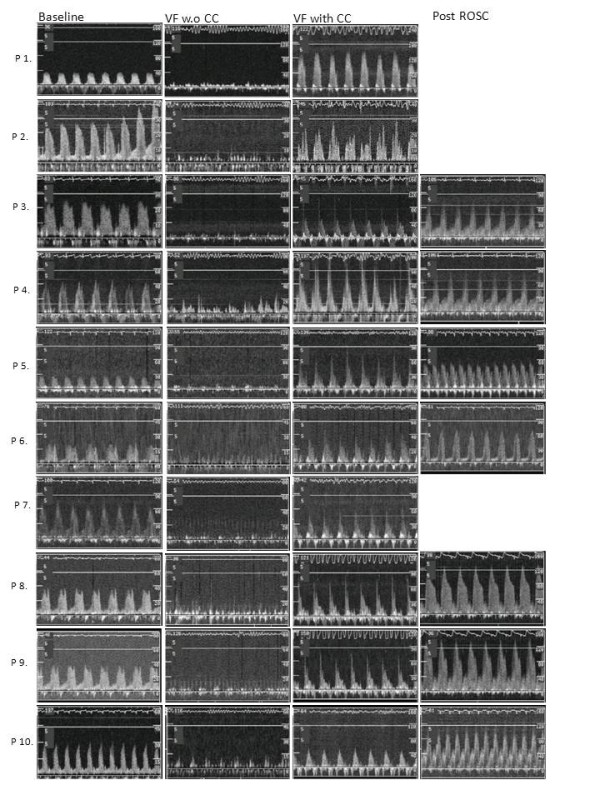
**Doppler curves from each of the four different experimental periods**. Doppler flow measurement from which the APV is calculated shown for all periods of the experiments and each pig (P n), baseline sinus rhythm (Baseline), untreated VF (VF without CC), VF during chest compressions (VF with CC) and post return of spontaneous circulation (Post ROSC). Note the difference in scale on the y-axis, which is due to automatic adjustments made by the FloMap monitor.

**Figure 2:**
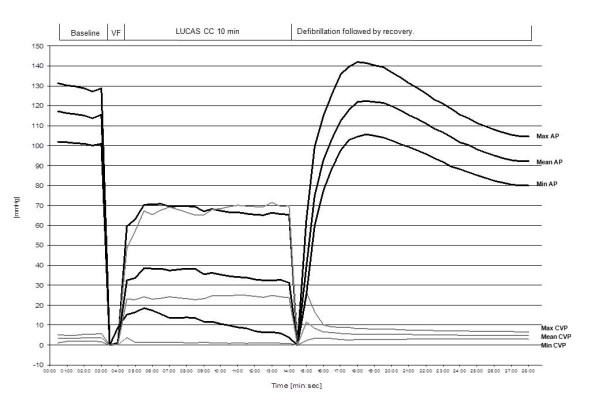
**Central Venous Pressure vs. Arterial pressure**. The development of minimum, mean and max (Min, Mean and Max) intra-thoracic aortic pressure and right atrial pressure during the total experimental period (28 min). Data are presented as the mean value of the 30 seconds periods of analyzed data, and each individual pigs data are time adjusted to same length for each period of the experiments.

**Figure 3:**
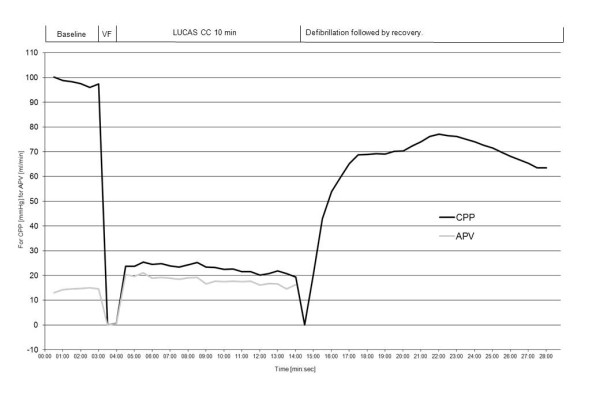
**Mean coronary perfusion pressure vs. Average Peak Velocity**. The development of the calculated mean coronary perfusion pressure (CPP in mmHg) during the total experimental period (28 min) and the development of coronary flow velocity (cm/s) during the experimental period. Coronary flow is not shown after ROSC due to technical problems after defibrillation. Data are presented as the mean value of the 30 seconds periods of analyzed data, and each individual pigs data are time adjusted to same length for each period of the experiments.

After baseline when all parameters were stable, especially flow velocity in the LAD, VF was induced with a 5-20 mA, 6 Hz and 30 V alternating current delivered to the epicardial surface via a needle electrode. Circulatory arrest was confirmed by a fall in AP, fall in Doppler flow velocity and an ECG showing VF upon which ventilation was stopped (Figure 1, 2 and 3). Following 60 seconds of VF, CPR was started using the electrically driven mechanical chest compression/decompression device LUCAS™2 (Jolife AB, Lund, Sweden) and ventilation was initiated at a rate of 10 inflations per minute. Continuous measurements of ECG, body temperature, AP, CVP and CPP, were performed. APV was documented by both a VHS-recorder and by digital recording. After ten minutes of CPR the pigs were defibrillated. If return of spontaneous circulation (ROSC) was not obtained after the first defibrillation, epinephrine 0.01 mg/kg was given in the central venous catheter and another defibrillation attempt made after 2 minutes, if persistent VF. Repeat doses of adrenaline and defibrillation attempts were performed as needed for a total of 3 times, with 2-minute intervals of chest compressions between each dose. After the third dose of epinephrine, CPR was continued for 2 minutes and then terminated. When ROSC was obtained, measurements continued 15 minutes with a ventilation rate of 20 breaths per minute with 100% oxygen in the respirator setting described above.

### Measurements

Arterial pressure and CVP were measured and the maximum, mean and minimum pressures were depicted. CPP was calculated as the difference between the thoracic intra-aortic pressure and the right atrial pressure in the end-decompression phase. The end of the decompression phase was defined between 0.1 - 0.05 seconds before the start of next compression [9,19]. Blood gases were drawn at baseline and after ten minutes of VF. APV, in the LAD was continuously measured and to evaluate the measurements, time periods which were visually free from noise and had a typical Doppler-curve like shape on the VHS-recording-tape where used for analysis (Figure 1) Time periods that were obviously artifacts were excluded. Arterial pressure, CVP and blood flow velocity signals were sampled 50 times/second and the mean value were recorded every 5 second during the whole experiment, using a computer supplied with a data acquisition system (Test Point, Capital Equipment Corporation, Bilerica, MA). Blood gas and electrolytes were analyzed directly after a sample had been obtained using a blood gas analyzer (ABL 505, Radiometer, Copenhagen, Denmark).

### Statistics

All data are presented as Mean ± standard deviation (SD). When analyzing and calculating the blood pressure curves and APV curves, these were analyzed as Mean ± SD of 30 second registration periods which then was divided in to 2-minute-intervals (e.g. 0-2 min, 2-4 min, 4-6 min, 6-8 min, 8-10 min) and a mean value for each period was calculated for each animal during the mechanical CC period. The difference in pressure and blood flow curves between baseline measurements during the VF-period and measurements during mechanical CCs were analyzed. The Mann-Whitney U test was used to determine differences between baseline measurements of APV and APV during mechanical CCs as well as for analyzing differences between blood gases. To test the null-hypothesis for correlation between APV and CPP, correlation z-test was used. Multiple continuous statistical comparisons were made between base line APV in each two minute periods as well as in the blood gas analysis; therefore we used the Bonferroni correction on all p-values. All analyses were carried out using StatView for Windows, Version 5.01 (SAS Institute Inc., SAS Campus Drive, Cary, NC, USA). A p-value < 0.05 was considered significant.

## Results

Seven animal regained ROSC following the first defibrillation attempt, two pigs required three defibrillation attempts to attain ROSC and two pigs did not obtain ROSC following defibrillation at the end of the 10 minute episode of VF of which one was excluded due to misplacement of the device before start of CCs. The baseline variables for the 10 pigs analyzed are shown in Table 1. The blood pressure and APV (APV noise free period was s: 0 - 2 min: 6 pigs = 66 ± 47 s, 2 - 4 min: 7 pigs = 114 ± 11 s, 4 - 6 min: 7 pigs = 99 ± 38 s, 6 - 8 min: 9 pigs = 68 ± 46 s, 8 - 10 min: 7 pigs = 84 ± 40 s) are shown in table 1, the statistical comparison revealed a significant (P < 0.0005) increase in coronary flow with mechanical CCs (CC-frequency 100 ± 1) compared to baseline (sinus rhythm, at a frequency of 97 ± 16) at each time interval, ranging between 12 and 39% increase (Table 1). Figure 1 shows the original Doppler curves from which APV was calculated during baseline, during the VF-period without CC, during the VF with mechanical CC and during the ROSC period indicating not only an instant increase in peak velocity but Doppler flow through the whole cyclic period of mechanical CCs. Arterial pressure and CVP during the total study period are presented in Figure 2. The progress of calculated CPP and the measured intracoronary APV is shown during the experimental period in Figure 3. Actual APV values from the velocity time integral could not be measured correctly during ROSC-period by the software in the Flomap machine which were likely caused by large repeated Doppler scale changes in response to a large reactive hyperemia combined with aliasing. However, there is a hyperemic period evident by increased APV and CPP that are shown in Figures 2 and 3 indicating that increased Doppler flow is evident (Figure 1).

**Table 1:** Measurements of central venous and arterial pressures, CPP and APV

Variable/time	Baseline	0-2 min	2-4 min	4-6 min	6-8 min	8-10 min
**Min AP [mmHg]**	101.1 ± 1.1	16.9 ± 2.3	14.1 ± 1.1	11.9 ± 1.2	8.5 ± 1.2	5.6 ± 1.3
**Mean AP [mmHg]**	115.7 ± 1.2	35.7 ± 3.3	38.0 ± 0.7	36.3 ± 1.4	33.8 ± 0.8	37.8 ± 1.3
**Max AP [mmHg]**	129.3 ± 1.5	65.9 ± 6.4	69.9 ± 0.8	68.0 ± 1.1	66.2 ± 0.9	65.6 ± 0.9
**Min CVP [mmHg]**	1.7 ± 0.5	1.9 ± 1.3	1.2 ± 0.2	1.1 ± 0.2	8.1 ± 0.2	1.0 ± 0.3
**Mean CVP [mmHg]**	3.5 ± 0.2	23.2 ± 1.7	23.7 ± 0.4	23.8 ± 0.8	24.8 ± 0.4	24.2 ± 0.6
**Max CVP [mmHg]**	5.2 ± 1.8	59.5 ± 8.4	67.9 ± 1.1	66.7 ± 1.7	69.7 ± 0.9	70.1 ± 1.2
**CPP [mmHg]**	98.0 ± 2.0	24.3 ± 1.4	24.1 ± 0.5	23.6 ± 1.3	21.5 ± 1.2	20.6 ± 1.5
**APV [cm/s]**	14.3 ± 1.0	20.0 ± 1.2	18.9 ± 0.5	17.8 ± 0.9	17.3 ± 0.9	16.0 ± 1.1

Baseline measurements and measurements during 0 - 2 min, 2 - 4 min, 4 - 6 min, 6 - 8 min and 8 - 10 min of mechanical chest compressions. AP = intrathoracic aortic pressure, CVP = Central Venous Pressure (right atrial pressure), CPP = calculated coronary perfusion pressure, APV = Average peak velocity. Data are presented as mean ± SD, n = 10.

**Figure 4:**
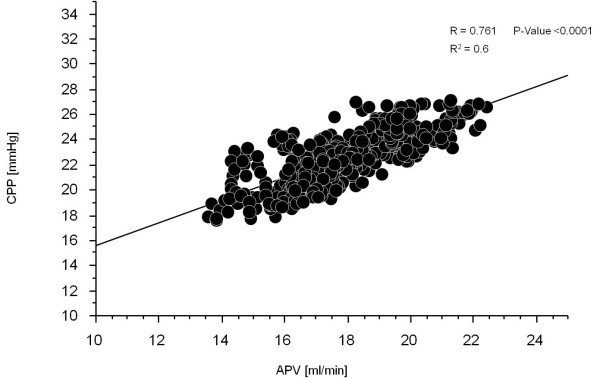
**Correlation of Coronary Perfusion Pressure and Average Peak Velocity**. Shows the correlation between calculated coronary perfusion pressure (CPP) and average peak velocity (APV) during the 10 min of mechanical chest compressions. To test the null-hypothesis for correlation between APV and CPP, correlation Z-test was used.

Correlation analysis between calculated CPP and APV was performed for the entire 10 min period of mechanical CCs. A significant correlation (R = 0.761, R2 = 0.6 and P-value < 0.0001) between the calculated CPP and APV during mechanical CCs was seen (Figure 4). Arterial blood gas values are shown in Table 2. There was a significant fall between base line and after ten minutes of mechanical CC in pH and Base Excess (BE) but Lactate was significantly elevated as well as glucose. The significant difference in pH, Lactate and glucose was persisting also following 20 min of ROSC, but BE stabilized and PO_2 _increased.

**Table 2:** Arterial blood gas measurements

Variable	Baseline		10 min		P-value	ROSC 20 min		P-value
	Arterial		Arterial			Arterial		
	**Mean**	**± SD**	**Mean**	**± SD**		**Mean**	**± SD**	

pH	7.350	± 0.026	7.257	± 0.060	0.012	7.287	± 0.050	0.014
PCO_2_	5.8	± 0.63	6.5	± 1.30	ns	6.4	± 0.83	ns
PO_2_	14.3	± 6.9	27.5	± 18.6	ns	48.75	± 17.7	0.042
ABE	-1.7	± 1.4	-6.0	± 1.4	0.002	-3.97	± 1.6	ns
Lactate	1.42	± 0.47	4.35	± 1.33	0.02	3.86	± 1.02	0.002
Hb	108	± 10	120	± 11	ns	114	± 10	ns
Na	137	± 2,1	136	± 2.7	ns	136	± 2.8	ns
K	3.96	± 0.24	4.59	± 0.89	ns	3.86	± 0.34	ns
Ca	1.33	± 0.04	1.35	± 0.06	ns	1.27	± 0.05	ns
Glucos	6.6	± 1.4	11.5	± 2.2	0.002	11.79	± 3.15	0.008

Measurements of blood gas, electrolytes, hemoglobin, hematocrit, lactate and glucose at base line and after ten minutes of mechanical chest compressions. Data are presented as Mean ± SD, n = 10. ns = not significant.

## Discussion

We found a significant correlation between CPP and APV during VF with circulation dependent on mechanical CCs. During ongoing mechanical CCs, APV was quantitatively equal or greater than baseline levels despite cardiac arrest, indicating that a mean CPP well above 20 mmHg might result in restored coronary blood flow.

Prolonged cardiac arrest in the cath-lab represents one of the biggest challenges to an interventional cardiologist. The recent introduction of mechanical chest compression devices may offer means of securing the initial circulation despite cardiac arrest. Several experiments have shown indirect evidence of good coronary circulation as measured by CPP and case studies have subjectively documented normalized coronary blood flow (TIMI III) during cardiac arrest during the use of mechanical chest compressions [1-3,20,21]. However, actual measurements of the coronary blood flow during cardiac arrest and ongoing mechanical chest compressions has been lacking. Reactive hyperemia may also play a role when assessing coronary blood flow with a Doppler flow wire, something which cannot be measured through evaluation TIMI flow during coronary angiograms or through CPP.

The initial measurements of APV and CPP at baseline were within the normal range for pigs. During VF without compressions both CPP and APV severely decreased. Then almost immediately following initiation of chest compressions both CPP and APV increased. APV increased to above baseline while CPP only increased to above 20 mmHg. Interestingly, APV and CPP correlate quite well during the VF phase although CPP is much lower than at baseline. During mechanical CCs arterial blood pressure was significantly lower than baseline resulting in an expected fall in CPP. However, APV was significantly higher compared to baseline during mechanical CCs and this was observed during the entire period of mechanical CCs. Following successful ROSC, CPP once again approaches baseline values while APV, after an initial increase, gradually decreases to return to the baseline values, but was still significantly higher than baseline at the end of mechanical CCs. This is a new finding as CPP has generally been assumed to correlate with coronary blood flow. How can APV be maintained close to baseline values while CPP remains significantly lower compared to baseline until after ROSC? One can speculate that the APV value is primarily driven by hyperemia caused by a post ischemic state and probably a release of a number of endogenous substances such as ATP and catecholamine's which is known to be extremely high in this situation in humans and animal resuscitation [22-24]. CPP however only correlates with a theoretical calculation of the coronary perfusion pressure which does not take into account a dilatation or constriction of the capillary bed of the myocardium which is of course of great importance for the actual coronary flow when measuring APV. Measurements of the proximal diameter of coronary arteries during angiograms indicate that there is very little difference in diameter of the coronary vessels during the baseline, VF and ROSC phases.

There was also an expected slight fall in pH as well as an increase in pCO_2 _tension, Base Excess, and lactate, probably because of the impaired circulation after ten minutes with ongoing VF and mechanical CCs. The elevation of CO_2 _may be the result of elevated CO_2 _production during ischemia and/or lowered excretion from the lungs. The elevation of blood glucose could be the result of endogenous produced epinephrine. The slight elevation in K^+ ^could be due to metabolic acidosis; however this remains speculative and needs to be further evaluated in future studies.

In this study we found CPP values and other physiological parameters that were comparable to earlier published data using a mechanical LUCAS device on pigs [6]. The CPP was high in this study as well. Judging from the visually observed TIMI-flow in the cath-lab, the mechanical chest compression device LUCAS seems capable of producing a TIMI-III flow in the cath-lab setting and corroborates earlier clinical findings [1-3,20,21].

### Limitations

In this study there are some limitations. First of all we used healthy juvenile pigs. As in all animal studies it is difficult to extrapolate these findings into human medicine in which cardiac arrest is commonly caused by an underlying heart disease such as an acute myocardial infarction or heart failure.

The length of the period with circulation performed with mechanical CCs in our study was limited to ten minutes which might be a limitation compared to clinical cardiac arrest cases. However, in our recently published study on humans with prolonged cardiac arrest in the cath-lab treated with mechanical CCs, mean treatment time among the survivors was 16 minutes (range 1 - 50 minutes) and in all patients 28 minutes (range 1 - 90 minutes) [2]. This indicates that coronary flow velocity is sufficient for extended periods of time in humans, when circulated with mechanical CCs.

Thus, as a resuscitation model this study does not mirror the true life situation for out of hospital cardiac arrest since we have optimal conditions with anaesthetized, intubated and otherwise fully controlled pigs in which we induced VF. However, the model can be more readily applied to, and conclusions drawn from scenarios in which patients suffer cardiac arrest in the cath-lab.

During ongoing mechanical chest compressions when evaluating coronary flow with APV, the measured curves were prone to movement artifacts from the mechanical CCs and the curves had to a large part be manually validated frame by frame from VHS recordings. During the hyperemia phase in the ROSC-period, it was not possible to perform reliable calculations of APV due to technical disturbances but there was a clear elevation of Doppler flow (Figure 1) indicating that APV reacted on the hyperemic phase as well. Furthermore, during the mechanical CCs, the coronary catheter had a tendency to further intubate the Left Main and LAD something which we have not seen in real patients during mechanical CCs. This was probably due to the angle of the catheters entry to the circulation from the left carotid artery; however this complication was continuously checked for and avoided but caused disturbances in the measurements of the APV.

## Conclusions

We conclude that there was a good correlation between CPP and APV which was highly significant, during mechanical CCs using an electrical driven LUCASTM2 device. In addition APV was even higher during mechanical CCs compared to baseline flow which also could be attributed to a combination of reactive hyperemia and endogenously produced epinephrine in the early phase of cardiac arrest. Thus, we have verified that mechanical CCs can, at minimum, re-establish coronary blood flow in non-diseased coronary arteries during cardiac arrest.

## Competing interests

Göran Olivecrona has received honorariums from Jolife AB and Medtronic Inc. for presenting case based lectures. Henrik Wagner has received honorariums from Jolife AB for presenting case based lectures. Bjarne Madsen-Härdig is an Employee of the Jolife AB Company. Stig Steen has received economical support for the research in cardiopulmonary resuscitation from Jolife AB. There are no other competing financial or non-financial interests.

## Authors' contributions

HW drafted the manuscript and has partaken in the animal experiments as well as acquisition and evaluation of data. BMH has partaken in the animal experiments and acquisition of data as well as critical evaluation of the manuscript. SS has partaken in the animal experiments and critical analysis of the manuscript. TS has partaken in the animal experiments as well as acquisition and evaluation of data he has also made critical evaluation of the manuscript and also helped with statistics. JH has been essential for critical evaluation of the manuscript. GO is responsible for concept and design as well as critical evaluation of the manuscript.

All authors have read and approved the final version of the manuscript.

## Authors' information

HW is an interventional cardiologist and physician on staff at the Department of Cardiology, Skåne University Hospital-Lund at the time of the conduction of the study. BMH is a former RN and PhD at the Department of Cardiology, Skåne University Hospital-Lund who is now employed by Jolife AB, Lund. SS is the Professor at the Department of Thoracic Surgery, Lund University. TS is an Associate Professor at the Department of Thoracic Surgery, Lund University. JH is an interventional radiologist and Associate Professor at Lund University. He is also a Consultant at the Department of Cardiology, Skåne University Hospital-Lund. GO is an interventional cardiologist and a Consultant at the Department of Cardiology, Skåne University Hospital-Lund.

## Pre-publication history

The pre-publication history for this paper can be accessed here:

http://www.biomedcentral.com/1471-2261/11/73/prepub
